# Supplementary material for: Surface-wave coupling in double Floquet sheets supporting phased temporal Wood anomalies
Source: Nanophotonics. 2022 Jul 11;11(15):3509–17. doi: 10.1515/nanoph-2022-0253 (PMC11501648; doi:10.1515/nanoph-2022-0253)
Supplement: Supplementary file 1 — Supplementary Material Details [file j_nanoph-2022-0253_suppl.docx]

Supplementary Material: Surface-Wave Coupling in Double Floquet Sheets Supporting Phased Temporal Wood Anomalies

Ya-Wen Tsai,^1^ Yao-Ting Wang,^2,3,5^ Emanuele Galiffi,^3^ Andrea Alù,^3,4^
and Ta-Jen Yen^1,6^

^1^ Department of Material Sciences and Engineering, National Tsing Hua University, Hsinchu 300, Taiwan.

^2^ Department of Mathematics, Imperial College London, London SW7 2AZ, UK

^3^ Photonics Initiative, Advanced Science Research Center, City University of New York, New York, NY 10031, USA

^4^ Physics Program, Graduate Center of the City University of New York, New York, NY 10016, USA

^5*^ ywang5@gc.cuny.edu

^6*^ [tjyen@mx.nthu.edu.tw](mailto:tjyen@mx.nthu.edu.tw)

1. Generalized transfer matrix of the double Floquet sheets structure

The derivation of the transfer matrix $\mathbf{M}$ is given in the main text, and can be written as follows

$\begin{matrix} \mathbf{M}_{2n\times2n}=\left[\mathbf{D}_{1,2}\mathbf{P}_{2}\left(d\right)\mathbf{D}_{2,3}\right]_{2n\times2n} \\ \text{ }=\left(\begin{matrix} & \vdots& \vdots& \vdots& \\ \cdots& M_{n+1,n+1} & M_{n+1,n} & M_{n+1,n-1} & \cdots\\ \cdots& M_{n,n+1} & M_{n,n} & M_{n,n-1} & \cdots\\ \cdots& M_{n-1,n+1} & M_{n-1,n} & M_{n-1,n-1} & \cdots\\ & \vdots& \vdots& \vdots& \end{matrix}\right) \end{matrix}$,

where the element $M_{p,q}$with ($p,q=\ldots,\text{ }n-1,\text{ }n,\text{ }n+1,\text{ }\ldots$) is a 2-by-2 matrix.

Here the matrices $\mathbf{D}_{12}$, $\mathbf{D}_{23}$, and $\mathbf{P}_{2}\left(d\right)$ are expressed as

$$\mathbf{D}_{j,j+1}=\left(\begin{matrix} & \vdots& \vdots& \vdots& \\ \cdots& X_{n+1} & Y_{n+1}^{-} & 0 & \cdots\\ \cdots& Y_{n}^{+} & X_{n} & Y_{n}^{-} & \cdots\\ \cdots& 0 & Y_{n-1}^{+} & X_{n-1} & \cdots\\ & \vdots& \vdots& \vdots& \end{matrix}\right),$$

with $\left(j=1,2\right)$, and

$\mathbf{P}_{2}\left(d\right)=\left(\begin{matrix} & \vdots& \vdots& \vdots& \\ \cdots& Z_{n+1} & 0 & 0 & \cdots\\ \cdots& 0 & Z_{n} & 0 & \cdots\\ \cdots& 0 & 0 & Z_{n-1} & \cdots\\ & \vdots& \vdots& \vdots& \end{matrix}\right)$.

In the Eqs. (S2) and (S3), the X, Y, and Z matrix elements are

,

,

and

with.

1. Convergence of transfer matrix for the double Floquet sheet system

In order to check the convergence against the 3-, 5-, and 7-harmonics cases, we truncate the matrices to the first three, five, seven Floquet modes as

$\left(\begin{matrix} 0 \\ E_{1x,0}^{+} \\ 0 \end{matrix}\right)=M_{3\times3}^{tra}\left(\begin{matrix} E_{3x,1}^{+} \\ E_{3x,0}^{+} \\ E_{3x,-1}^{+} \end{matrix}\right)=\left(\begin{matrix} m_{11} & m_{12} & m_{13} \\ m_{21} & m_{22} & m_{23} \\ m_{31} & m_{32} & m_{33} \end{matrix}\right)\left(\begin{matrix} E_{3x,1}^{+} \\ E_{3x,0}^{+} \\ E_{3x,-1}^{+} \end{matrix}\right)$,

$\left(\begin{matrix} 0 \\ 0 \\ E_{1x,0}^{+} \\ 0 \\ 0 \end{matrix}\right)=M_{5\times5}^{tra}\left(\begin{matrix} E_{3x,2}^{+} \\ E_{3x,1}^{+} \\ E_{3x,0}^{+} \\ E_{3x,-1}^{+} \\ E_{3x,-2}^{+} \end{matrix}\right)=\left(\begin{matrix} m_{11} & \cdots& m_{15} \\ \vdots& \ddots& \vdots\\ m_{51} & \cdots& m_{55} \end{matrix}\right)\left(\begin{matrix} E_{3x,2}^{+} \\ E_{3x,1}^{+} \\ E_{3x,0}^{+} \\ E_{3x,-1}^{+} \\ E_{3x,-2}^{+} \end{matrix}\right)$,

$\left(\begin{matrix} 0 \\ 0 \\ 0 \\ E_{1x,0}^{+} \\ 0 \\ 0 \\ 0 \end{matrix}\right)=M_{7\times7}^{tra}\left(\begin{matrix} E_{3x,3}^{+} \\ E_{3x,2}^{+} \\ E_{3x,1}^{+} \\ E_{3x,0}^{+} \\ E_{3x,-1}^{+} \\ E_{3x,-2}^{+} \\ E_{3x,-3}^{+} \end{matrix}\right)=\left(\begin{matrix} m_{11} & \cdots& m_{17} \\ \vdots& \ddots& \vdots\\ m_{71} & \cdots& m_{77} \end{matrix}\right)\left(\begin{matrix} E_{3x,3}^{+} \\ E_{3x,2}^{+} \\ E_{3x,1}^{+} \\ E_{3x,0}^{+} \\ E_{3x,-1}^{+} \\ E_{3x,-2}^{+} \\ E_{3x,-3}^{+} \end{matrix}\right)$,

where $m_{p,q}$ with ($p,q=\ldots,\text{ }n-1,\text{ }n,\text{ }n+1,\text{ }\ldots$) is the element of the transmitted matrix $M^{tra}$.

By solving the reduced transfer matrices in Eqs. (S7-S9), the approximate solutions of the transmitted amplitudes with time modulation are then obtained. Suppose that the incident wave is from the top upon the DFSs with the transmission coefficient for the fundamental harmonics denoted by T, it can be shown that the transmission coefficient of the three cases are given by the elements of $M_{3\times3}^{tra}$, $M_{5\times5}^{tra}$, $M_{7\times7}^{tra}$ as,

$\text{T}_{3-harmonics}=\frac{E_{3x,0}^{+}}{E_{1x,0}^{+}}=\frac{\det\left[M_{3\times3}^{tra}\left(2\right)\right]}{\det\left(M_{3\times3}^{tra}\right)}$,

$\text{T}_{5-harmonics}=\frac{E_{5x,0}^{+}}{E_{1x,0}^{+}}=\frac{\det\left[M_{5\times5}^{tra}\left(3\right)\right]}{\det\left(M_{5\times5}^{tra}\right)}$,

$\text{T}_{7-harmonics}=\frac{E_{7x,0}^{+}}{E_{1x,0}^{+}}=\frac{\det\left[M_{7\times7}^{tra}\left(4\right)\right]}{\det\left(M_{7\times7}^{tra}\right)}$,

where $M^{tra}\left(\text{i}\right)$ is the transmitted matrix formed by replacing the i-th column of $M^{tra}$ by the column vector $\left(\cdots,0,0,E_{1x,0}^{+},0,0,\cdots\right)^{T}$. The transmittance spectrum of the different number of harmonics cases in Fig. S1 can be calculated by Eqs. (S10)-(S11). Fig. S1 shows that the DFS system has good agreement under the 5- (red dashed) and 7-harmonics (green dot) cases, meaning both cases are well converged. For the 3-harmonic case, the spectrum discrepancy comparing to 5 or 7 harmonic cases is less than 1%, which is numerically valid in investigating the DFS system. Therefore, the 3-harmonics case, rather than the better converging case, is chosen to be an approximated model in the main text because of its computational efficiency.


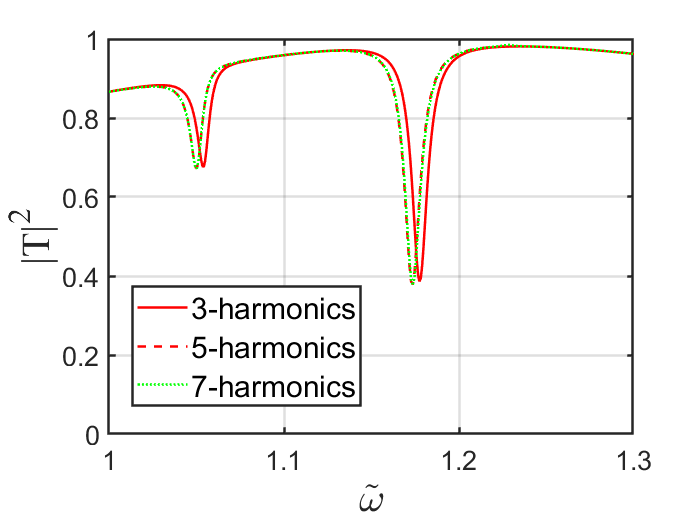


Figure S1: Transmittance spectra of the DFS structure for three different number of harmonics cases with time modulation $\text{(}\alpha=0.15\text{)}$ at fixed in-plane wavevector $\tilde{k}_{x}=0.75$. The following parameters are used: $\tilde{d}=2$, $\varepsilon_{1}=\varepsilon_{2}=\varepsilon_{3}=1$, $\tilde{\Omega}\approx0.6$, and $\tilde{\gamma}\approx0.01$.

1. The FWHM values of three Gaussian pulses

In Fig. 4 of the main text, the FWHM of Gaussian pulses plays a very important role in demonstrating the switching capability of the DFS system. To check the FWHM of each pulse, we simulate the pulse traveling through air by using the time-dependent transient electromagnetic waves (temw) module in COMSOL. The four boundaries of the simulation window in Fig. S2 are set as scattering boundary conditions (SBCs), and the gray area is air. A Gaussian pulse is injecting from the top side [blue line in Fig. S2] as the incident wave. The expression for the relative magnetic field of a Gaussian pulse is

$H_{z}\left(y\right)=H_{0}e^{-\left(t-t_{0}\right)^{2}/\Delta t^{2}}e^{-\left(x-x_{0}\right)^{2}/\Delta x^{2}}\cos\left(k_{y}y-\omega_{0}t\right)$,

where $k_{y}y=\omega_{0}\left[\cos\left(\theta\right)x+\sin\left(\theta\right)z\right]/c$ and $\omega_{0}$ is the carrier frequency. All relevant parameters of the three Gaussian pulses are listed in table S1. In addition, in Fig.S2 the fixed red dot at the blue boundary represents a “probe point” in COMSOL. Thus, from that point we acquire the transient data of a complete Gaussian pulse. A fast Fourier transformation (FFT) is then applied to the pulse obtained in the previous step in order to evaluate the FWHM of the pulse. The resulting pattern of the three pulses is shown in Fig. S3. In Fig. S3, the FWHM values of two on-resonance pulses and one off-resonance pulse are 0.045, 0,045, and 0.3, respectively.


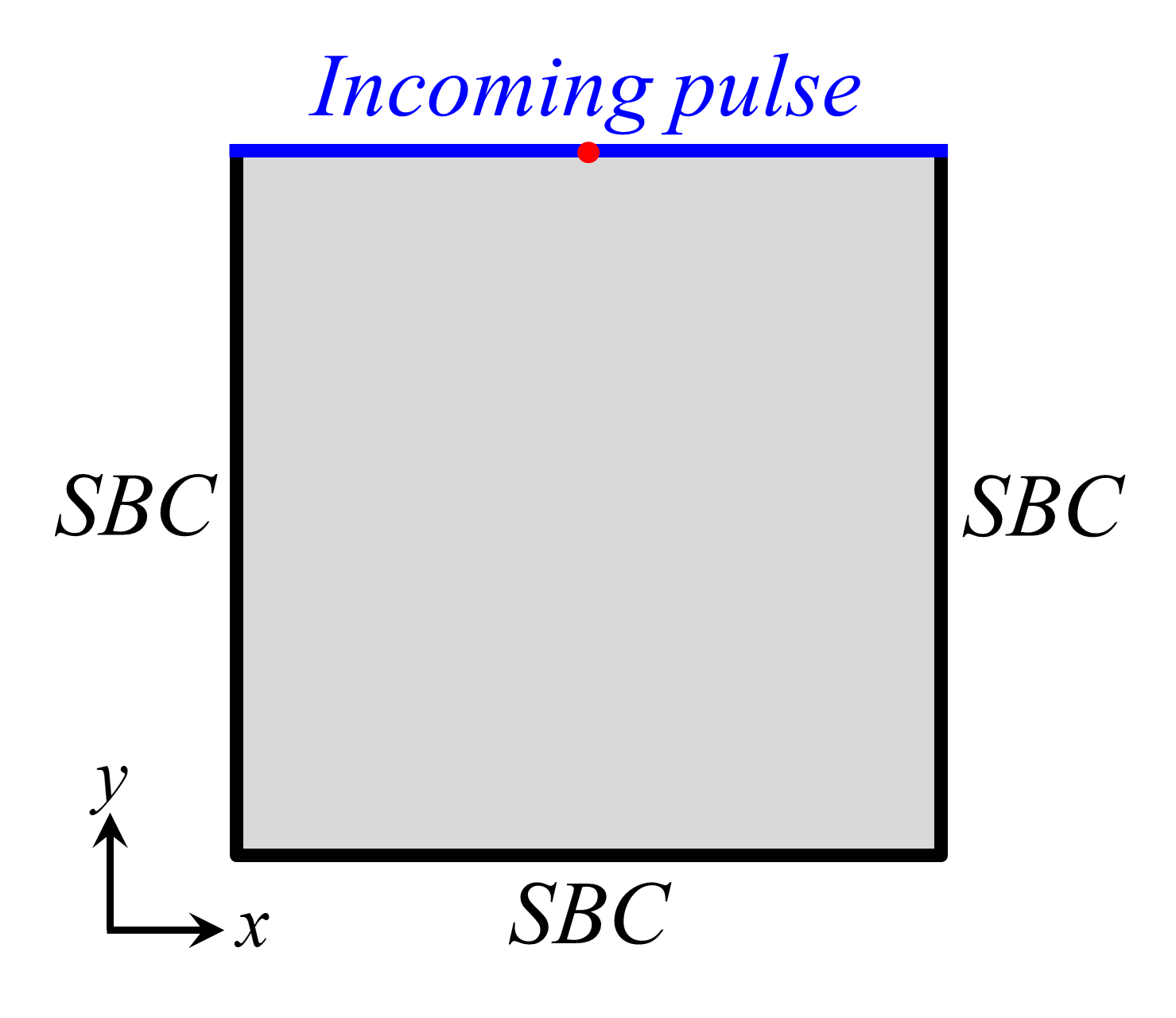


Figure S2: The simulation window.

Table S1: Parameters of three Gaussian pulses

|  | Gaussian pulses | | |  |
| --- | --- | --- | --- | --- |
| Symbol | On-resonance pulse with $\tilde{\omega}_{0,-}$ | On-resonance pulse with $\tilde{\omega}_{0,+}$ | Off-resonance pulse with $\tilde{\omega}_{0,mid}$ | Description |
| $\tilde{\omega}_{0}$ | 1.056 | 1.176 | 1.116 | Carrier frequency |
| $\tilde{k}_{x}$ | 0.75 | 0.75 | 0.75 | In-plane wavevector |
| $\tilde{k}_{y}$ | 0.743 | 0.906 | 0.826 | Out-of-plane wavevector |
| $\theta$ | $45^{\circ}$ | $39.6^{\circ}$ | $42^{\circ}$ | Incident angle |
| $\Delta\tilde{t}$ | 3.014 | 2.707 | 0.285 | Temporal width of pulse |
| $\Delta\tilde{x}$ | 23.8 | 21.37 | 5.63 | Spatial width of pulse |
| $\tilde{t}_{0}$ | 266 | 266 | 44.33 | Temporal delay of pulse |


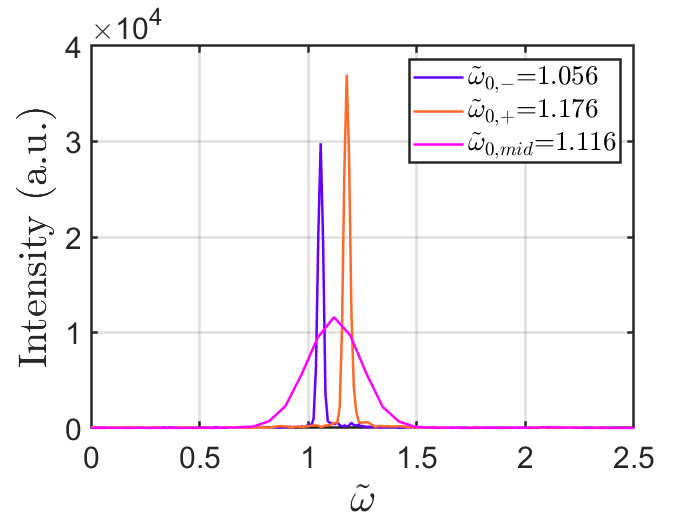


Figure S3: The FFT result of the three Gaussian pulses with different carrier frequencies resonant, which are $\tilde{\omega}_{0,-}\approx1.056$ (purple curve),$\tilde{\omega}_{0,+}\approx1.176$ (orange curve), and $\tilde{\omega}_{0,mid}\approx1.116$ (pink curve).

1. A possible implementation demonstration

In this section, we purpose an experimental implementation in a DFS device in high-quality graphene. In Fig. S4(a), we begin by calculating the dispersion relation with the interlayer gap $d\approx0.17$ mm and reveal the coupling process of the system. Given modulation frequency $f_{m}\approx0.16\text{ THz}$, we then calculate the reflection spectrum by varying the phase difference between the two sinusoidal modulations from 0 to $2\pi$ and the incident frequency $f$ from 0.48 THz to 0.6 THz, for a fixed incoming wavevector $k_{x}=8.87\text{ }\text{rad}/\text{mm}$, as shown in Fig. S4(b). The results show that the strong coupling efficiency to either one of the surface modes can be achieved when or $\pi$. Fig. S4(c) shows the reflection spectra of the DFS system as phase modulation and $\pi$, respectively. The phase-modulated case with ($\pi$) exhibit one clear reflection dip at $f_{0+}\approx f_{SW+}+f_{m}\approx0.57\text{ THz}$ ($f_{0-}\approx f_{SW-}+f_{m}\approx0.515\text{ THz}$), indicating that $40\text{ }\%$ ($35\text{ }\%$) of reflection power is converted to the symmetric (anti-symmetric) mode. In addition, Fig. S4(d) shows that the coupling efficiency of the excited mode increases when the modulation amplitude increases. In the main text, we use the $\alpha=0.15$ case as an example.


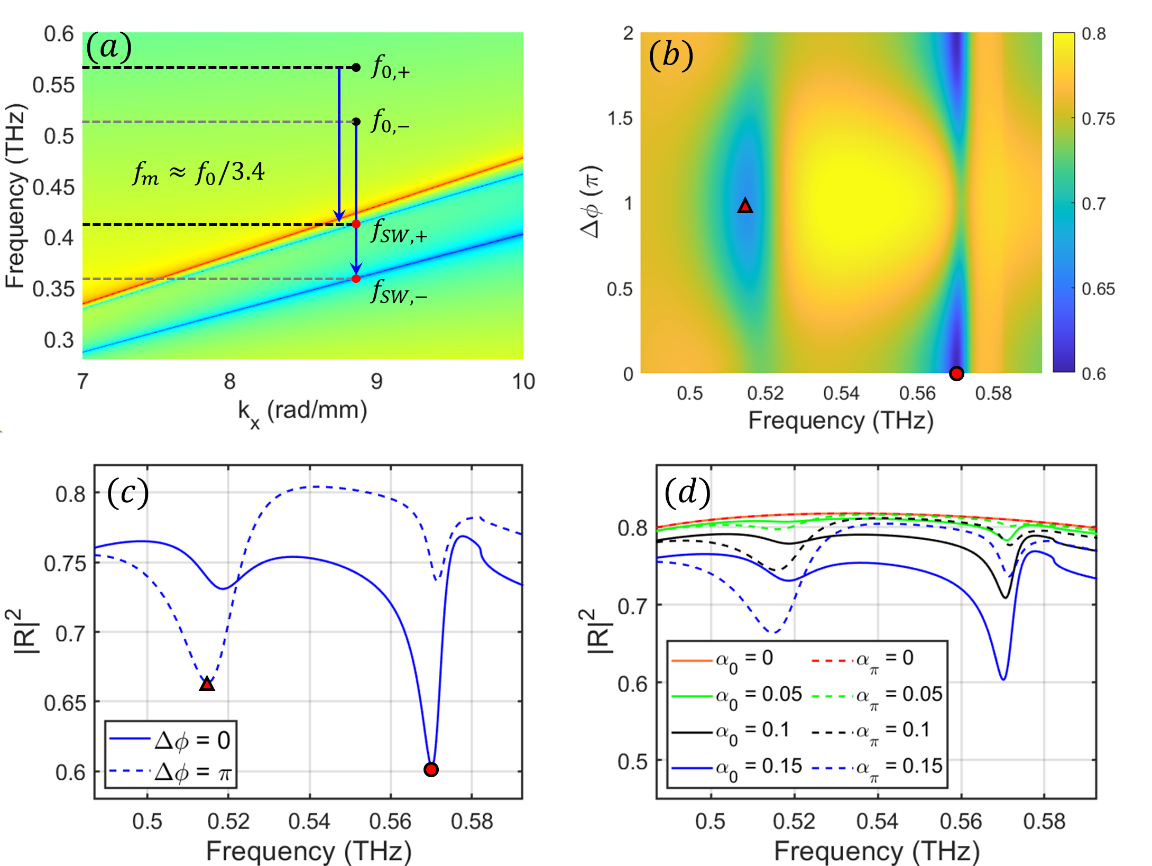


Figure S4: Numerical calculated results of the DFS device. (a) Dispersion relation for surface mode with the interlayer gap between two graphene layers $d\approx0.17$mm. The corresponding parameters of the graphene layers are $E_{F,0}=0.3\text{ eV}$, , $f_{m}=\Omega/2\pi\approx0.16\text{ THz}$, $v_{F}=9.5\times10^{7}\text{cm}/\text{s}$, $m=100\times10^{3}\text{cm}^{2}/\left(\text{V}\cdot\text{s}\right)$, and $\gamma=ev_{F}^{2}/\left(mE_{F}\right)\approx0.3$THz. (b) Contour plots of the reflection spectra as a function of incident frequency $f$ and phase difference . The red triangle and circle markers highlight the maximum values of the reflection which are the odd mode and even mode. (c) Reflection spectra of the DFS device with (blue solid line) and $\pi$(blue dashed line). The positions of the red triangle and circle markers represent the two resonance frequencies in (b). (d) Reflection spectra results for the DFS device with different values of the modulation strength from 0 (red), 0.05 (green), 0.1 (black), to 0.15 (blue). The cases of solid line and dashed line are shown for and , corresponding to the even mode excitation and odd mode excitation.
